# Older adult falls prevention behaviors 60 days post-discharge from an urban emergency department after treatment for a fall

**DOI:** 10.1186/s40621-017-0114-y

**Published:** 2017-06-19

**Authors:** Kalpana Narayan Shankar, Nicole J. Treadway, Alyssa A. Taylor, Alan H. Breaud, Elizabeth W. Peterson, Jonathan Howland

**Affiliations:** 1 Department of Emergency Medicine, Boston University Medical Center and Boston University School of Medicine, One Boston Medical Center Place, Dowling 1 South, Boston, MA 02118 USA; 2 Boston Medical Center Injury Prevention Center, One Boston Medical Center Place, Boston, MA 02118 USA; 3 Department of Occupational Therapy, University of Illinois at Chicago, 1919 W. Taylor St, Chicago, IL 60612 USA

**Keywords:** Falls older adults, Falls prevention, Emergency departments, Recidivism

## Abstract

**Background:**

Falls are a common and debilitating health problem for older adults. Older adults are often treated and discharged home by emergency department (ED)-based providers with the hope they will receive falls prevention resources and referrals from their primary care provider. This descriptive study investigated falls prevention activities, including interactions with primary care providers, among community-dwelling older adults who were discharged home after presenting to an ED with a fall-related injury.

**Methods:**

We enrolled English speaking patients, aged ≥ 65 years, who presented to the ED of an urban level one trauma center with a fall or fall related injury and discharged home. During subjects’ initial visits to the ED, we screened and enrolled patients, gathered patient demographics and provided them with a flyer for a Matter of Balance course. Sixty-days post enrollment, we conducted a phone follow-up interview to collect information on post-fall behaviors including information regarding the efforts to engage family and the primary care provider, enroll in a falls prevention program, assess patients’ attitudes towards falling and experiences with any subsequent falls.

**Results:**

Eighty-seven community-dwelling people between the ages of 65 and 90 were recruited, the majority (76%) being women. Seventy-one percent of subjects reported talking to their provider regarding the fall; 37% reported engaging in falls prevention activities. No subjects reported enrolling in a fall prevention program although two reported contacting falls program staff. Fourteen percent of subjects (*n*=12) reported a recurrent fall and 8% (7) reported returning to the ED after a recurrent fall.

**Conclusions:**

Findings indicate a low rate of initiating fall prevention behaviors following an ED visit for a fall-related injury among community-dwelling older adults, and highlight the ED visit as an important, but underutilized, opportunity to mobilize health care resources for people at high risk for subsequent falls.

**Electronic supplementary material:**

The online version of this article (doi:10.1186/s40621-017-0114-y) contains supplementary material, which is available to authorized users.

## Background

Falls among older adults are a common and serious public health problem that can cause debilitating, sometimes fatal, injuries and affect psychosocial status and quality of life. Each year, a third of those 65 year of age or older fall; among this age group falls are the leading cause of fatal and non-fatal injuries (Important Facts about Falls | Home and Recreational Safety | CDC Injury Center 2017). In 2015, 2.5 million older adults were treated in emergency departments (EDs) for non-fatal fall-related injuries and more than 734,000 of these patients were hospitalized. In that year, the direct medical costs for older adult falls ranges between $31.3 billion to $36.8 billion, both adjusted for inflation for 2015 (Important Facts about Falls | Home and Recreational Safety | CDC Injury Center 2017; Deileman et al. 2016). Even when falls do not require medical attention, the experience can result in fear of falling, which can be psychologically disabling (Bell et al. 2000), and lead to future falls through physical deconditioning (Delbaere 2004; Howland et al. 1998).

Low cost, low-tech community-based interventions that are evidence-based for falls prevention have been developed (Gillespie et al. 2012). These programs typically result in 25–30% reductions in 1-year post-program falls (Gillespie et al. 2012). They are increasingly deployed throughout the nation and are most often offered by public and private organizations that serve older adults. Program recruitment usually occurs through direct marketing to older adults, rather than through referrals from healthcare providers. Nonetheless, falls prevention programs may eventually be integrated with the healthcare system as physicians become more engaged in falls risk assessment for their older patients and older adults become more aware that falls risk can be reduced. New instruments, such as the STEADI (Stop Elderly Accidents, Deaths, and Injuries) Toolkit, developed by the Centers for Disease Control and Prevention, can facilitate older adult falls-risk assessment by healthcare providers (Stevens 2013). Moreover, as healthcare reform continues to promote evidence-based models that can prevent acute care visits, physician groups and hospitals will likely become more interested in providing risk-reduction programs.

In addition to randomized trials demonstrating the effectiveness of community-based interventions for reducing falls and/or fear of falling, recent studies have also shown these programs to be cost-effective. The Centers for Medicare and Medicaid Services (2013) conducted a retrospective cohort study evaluating *A Matter of Balance* (MOB), a program developed to reduce fear of falling and increase mobility in older adults (Tennstedt et al. 1998). Compared to matched controls, older adults who had participated in the MOB program had, during the post-participation year, significantly lower total health care costs (Centers for Medicare and Medicaid Services 2013). A recent study estimated the net benefit and return on investment (ROI) of three falls prevention programs. *Otago*, a program targeting frail older adults and delivered in the home by a physical therapist or other healthcare provider, had a 1-year net benefit of $121.85 and a ROI of 36% for each dollar invested. *Tai Chi: Moving for Better Balance*, a group program for enhancing strength and balance, had a 1-year net benefit of $529.86 and a ROI of 509% for each dollar invested. *Stepping On*, combining community-based group sessions with follow-up home visits by a healthcare provider, had a 14-month net benefit of $134.37 and a ROI of 64% for each dollar invested (Carande-Kulis et al. 2015).

Older adults who have experienced a fall are at increased risk for future falls (Tiedemann et al. 2012; Ambrose et al. 2013; Close et al. 2011; Russell et al. 2010; Carpenter et al. 2009; Rubenstein LJosephson 2006). With respect to ED recidivism following discharge for a fall-related injury, Tiedemann et al. (2012) found that 31% of community-dwelling older adults discharged from EDs following care for a fall-related complaint fell again within six months and that 62% of these post-discharge falls were injurious. Liu et al. (2015) noted that 36% of patients return to the ED for any reason or die within 1 year of a fall. Close et al. (2011) found that 35.4% of older adults who presented at an urban ED for a fall-related injury returned to the ED for a subsequent fall injury at least once during the following year. In a trial of a multifactorial falls prevention program for older adult patients who presented at an ED with a fall-related injury and were discharged home, 18% of patients presented again for treatment of a fall-related injury (Russell et al. 2010).

The fall event should trigger a health system response to mitigate the risk of subsequent falls. Several studies, however, indicate that many older adults discharged from EDs for a fall-related injury are not referred for, and do not receive, post-discharge comprehensive fall prevention care (Naughton et al. 2012; Salter et al. 2006; Donaldson et al. 2005). A study profiling older adult fall patients treated at an ED (Naughton et al. 2012), found that one third were discharged without evidence of a referral. Salter et al. (2006) conducted 6-month follow-up with older adults discharged home after treatment for a fall injury at an urban ED. Participants’ mean fall risk score increased significantly over the 6-month follow-up period and only 2 (3.7%) received post-discharge care consistent the American Geriatric Society Guidelines (American Geriatric Society and British Geriatric Society 2010). In a companion study, Donaldson et al. (2005) followed older adult women 18-month post-discharge from an ED for a fall injury and found that only 32% had been referred to their family physician and only 24% referred to physiotherapy. However Salter et al. (2006) and Donaldson et al. (2005) were limited by their sampling frame and Donaldson et al. (2005) was further limited by recall bias due to the length of their follow-up.

To better understand older adults’ experience of engaging in fall prevention behaviors after a fall-related ED visit, we investigated post-fall behaviors by community-dwelling older adults during the 60-days following an ED visit for a fall or fall-related injury.

## Methods

### Screening & enrollment

Between the hours of 7 am to 11 pm, a study Research Assistant (RA) monitored electronic medical records (EMRs) documenting in real time patients presenting at a large urban level one trauma center. When patients were identified as having a fall-related injury per the EMR (chief complaint and/or triage summary and/or patient care timeline) and being ≥ 65 years of age, the RA approached the attending physician to determine if the patient met eligibility criteria, including confirmation that the presenting problem was fall-related, that the patient was community-dwelling and would be discharged home. If these criteria were met, the RA obtained patient consent for enrollment along with contact information for a follow-up phone call. Upon discharge, those enrolled in the study received a flyer describing a local evidence-based program designed to reduce fear of falling and increase activity levels as this was part of the standard discharge instructions given to all elder falls patients who were discharged from the ED. These flyers were also visible and available to all ED patients in the designated common areas.

### Eligibility

#### Inclusion/exclusion criteria

Patient were eligible for the study if they: (1) were at least 65 years of age; (2) were living independently in the community at a permanent address; (3) were presenting at the ED as a consequence of an unintentional fall-related injury; (4) were discharged from the ED, without inpatient admission; (5) had decision making capacity as determined by the ability to comprehend the study and consent procedures in English and subsequently by answers to post-consent questions about the study and consent content (Karlawish 2007; Appelbaum 2007; Grisso et al. 1997). (6) had access to a telephone at home; and (7) had adequate hearing to routinely use the telephone, as determined by a query by the RA *(e.g., “Do you have trouble hearing when you use the phone?”)*.

Patients were excluded if they were: (1) combative or in police custody (as per ethical considerations); (2) deemed to be intoxicated with alcohol, narcotics or sedatives (as determined by clinical staff); (3) undomiciled after discharge; (4) unable to consent to the study; (5) unable to name a locator person who would know how they can be reached; (6) wheelchair bound; (7) referred by clinical staff for investigation of possible elder neglect or abuse; (8) refusing permission for follow-up contact; (9) legally blind; (10) non-English speaking; or, (11) unable to provide a phone number for follow-up contact.

Some patients were consented based on the understanding that they were to be discharged home but were subsequently reevaluated and admitted to the hospital. These patients became ineligible for the study.

### Follow-up

Sixty days post-discharge, the RA attempted to contact the participant by phone. Up to 18 calls were made and 5 messages left before the participant was considered to be lost to follow-up. If the participant was reached, the RA administered the follow-up questionnaire. On average, the questionnaire took about 5-7 min to complete.

### Measures

#### Participant characteristics

We collected information on participants’ age, sex, race/ethnicity, ED admission date, ED discharge date, Medicaid status, and zip code of residence.

#### Family and provider engagement with falls prevention

To assess post-discharge the patient’s engagement with family and their provider regarding their fall and any falls prevention interventions, we used an 8-item questionnaire we developed for this study (Table 1).

**Table 1:** Patient assessment questionnaires

*Family and Provider Engagement with Fall Prevention:* Response options for all questions were *Yes or No*, except for Question 4, for which the response was number of days.	
(1) *Since you were discharged on [date] for your fall injury, have you spoken to a healthcare provider about the fall you had?;*	
(2) *During the last two months, have you talked to your healthcare provider(s) about things you can do to reduce your chance of falling?*;	
(3) *During the last two months, have you talked with your healthcare provider(s) about how your medications might influence your chance of falling?;*	
(4) *Can you recall how many days after you were discharged from the emergency department on [date] that you spoke to your healthcare provider about the fall you had?*;	
(5) *During the last two months, have you talked with your pharmacist about how your medications might influence your chance of falling?*;	
(6) *During the last two months, have you talked with your healthcare providers, including eye doctor or optometrist, about how your vision might influence your chance of falling?*;	
(7) *During the last two months, have you talked with family members about things you can do to reduce your chance of falling?*;	
(8) *During the last two months, have you talked with friends about things you can do to reduce your chance of falling?*	
*Falls Prevention Program Participation*	
(1) *During the last two months, have you contacted or attempted to contact a falls prevention program offered in your community?* If participants answered *Yes*, they were asked Question 2; if they answered *No*, they answered Question 3.	
(2) *During the last two months, have you participated in any community-based falls prevention programs or exercise programs, such as Tai Chi, Stepping On, or Matter of Balance?* If they answered *Yes*, they were asked	
(a) *What was the name of the program?*	
(b) *Where did the program meet;*	
(c) *When did the program start?* (M/D/Y);	
(d) *When did the program end?* (M/D/Y);	
(e) *Are you going to the program now?* (*Yes, No*);	
(f) *Usually, for how many minutes does the program session last* (# of MINS);	
(3) *How likely is it that you will participate in a community-based falls prevention program in the next year? Would you say it was* (response options: *very unlikely; somewhat unlikely; neither likely nor unlikely, somewhat likely, very likely*).	
*Post discharge falls assessment*	
(1) *Since you were enrolled in this study two months ago, on (date), have you had any additional falls?* (*Yes*/*No*);	
(2) *About how many times have you fallen in the last two months since (date)*? (Total number of falls);	
(3) *Did any of the falls you had in the last two months require medical attention?* (*Yes/No*);	
(4) *Was the medical attention you received at an emergency department?* (Yes/No); (5) *Number of times they had been treated for fall injury there since enrollment.*	

#### Falls prevention program participation

We also used a brief questionnaire to determine whether participants had engaged, or planned to engage in, post-discharge in falls prevention programs (Table 1).

#### Post-discharge falls assessment

We asked several questions to ascertain subjects’ fall experiences after the ED visit leading to study enrollment (Table 1).

#### Receipt of brochure

Participants were asked whether they recalled receiving a brochure on a falls prevention program when they were discharged from the ED (Yes/No).

### Data analysis

The aim of this study was descriptive, documenting the frequency with which patients discharged from an ED following treatment for fall-related injuries engaged in falls prevention activity during the subsequent 60 days. Accordingly, a power analysis was not conducted. Nevertheless, statistical procedures were conducted to examine differences by gender. For these analyses, Chi Square was used for categorical variables and non-paired *t*-test for continuous variables.

## Results

**Fig. 1:**
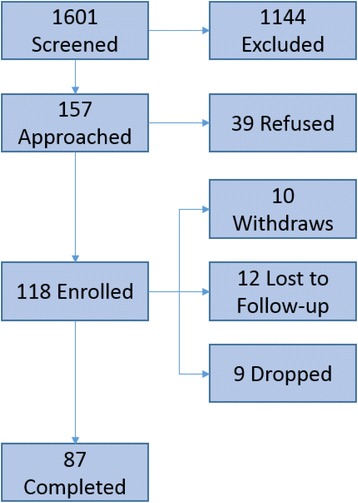
Enrollment Flow Diagram

One hundred eighteen patients were enrolled, of which 87 (74%) completed the follow-up questionnaire. Fig. 1 presents enrollment/follow-up flow, including number of patients screened for eligibility, number offered participation, number enrolled, and number followed-up.

Participant characteristics are shown in Table 2.

**Table 2:** Participant characteristics

Variable	All participants (*n* = 87)
Mean age	72 years
Age range	65-90 year
% Male	24%
% White	39%
% Black/African American	54%
% Asian	1%
% Hispanic/Latino	5%
% Other	1%
% Medicaid +-/Medicare	37%
% Private +/- Medicare	41%
% Medicare only	22%

### Help-seeking and communications

Seventy-one percent of the subjects (67% of males, 71% of females) reported talking to a healthcare provider about their fall-related ED visit that led to enrollment in the study. On average, participants spoke to their healthcare providers about their fall events 11 days (13 for males; 10 for females) post discharge. Thirty-seven percent (29% of males, 39 females) reported talking to their healthcare provider about things to do to reduce falls risk. Twenty-two percent (10% of males, 24% of females) reported talking to their healthcare provider about how medications can affect falls risk. Eleven percent (5% of males, 14% of females) reported talking to their pharmacist about how medications can affect falls risk. Fifteen percent (10% of males, 17% of females) reported talking to their healthcare provider, including ophthalmologist or optometrist, about how vision problems can affect falls risk. Forty-six percent (29% of males, 52% of females) reported % talking to family members about things to do to prevent falls and 37% (24% of males, 41% of females) reported talking to friends about things to do to prevent falls (Table 3).

**Table 3:** Post-Discharge Falls Prevention Behaviors by Gender

Variable^a^	Males (*n*= 21)	Females (*n* = 66)	All participants (*n* = 87)
Spoken to provider about your fall	14 (67%)	47 (7%)	62 (71%)
Spoken to provider about falls prevention	6 (29%)	26 (39%)	32 (37%)
Spoken to provider about medications	2 (10%)	16 (24%)	19 (22%)
Number of days post-discharge to speak with provider regarding the fall *(mean)*	13	10	11
Spoken to pharmacist about medications	1 (5%)	9 (14%)	10 (11%)
Spoken to providers about vision	2 (10%)	11 (17%)	13 (15%)
Spoken to family about falls prevention	6 (29%)	34 (52%)^b^	40 (46%)
Spoken to friends about falls prevention	5 (24%)	27 (41%)^b^	32 (37%)

^a^ Default answer is “yes”; ^b^ 1 female participant did not answer this question

Participants’ engagement in falls prevention programs is shown in Table 3

#### Falls prevention program participation

Two percent (0% of males, 3% of females) reported that they had attempted to contact a falls prevention program in their community.

None of the participants reported having attended a community-based falls prevention program, such as Matter of Balance or Tai Chi, although, 42% (67% of males; 35% of females) said that it was somewhat or very likely that they would participate in a community-based falls prevention program during the next year (Tables 4 and 5).

**Table 4:** Participation in falls prevention programs

Variable	Males (*n* = 21)	Females (*n* = 66)	All Participants (*n* = 87)
Contacted/attempted to contact falls prevention program	0 (0%)	2 (3%)	2 (2%)
Participated in falls prevention program (e.g. MOB, Tai Chi)	0	0	0
*How likely you will participate in falls prevention program in next year?*			
Very unlikely	3 (14%)	28 (42%)	31 (36%)
Somewhat unlikely	2 (10%)	12 (18%)	14 (16%)
Neither likely nor unlikely	2 (10%)	3 (5%)	5 (6%)
Somewhat likely	10 (48%)	11 (17%)	21 (24%)
Very likely	4 (19%)	12 (18%)	16 (18%)

**Table 5:** Subsequent falls

Variable	Males	Females	All participants
Subsequent falls post-discharge *n* (%)	3 (14%)	9 (17%)	12 (14%)
Mean # falls post-discharge	3.67	1.8	2.75
Post-discharge falls require medical attention *n* (%)	1 (5%)	6 (9%)	7 (8%)
Medical attention at Emergency Department *n* (%)	1 (5%)	6 (9%)	7 (8%)

#### Subsequent falls

Fourteen percent of males (3/21) and females (9/66) reported experiencing at least one subsequent fall during the 60-day post discharge period. Of all participants, 5% (1/21) of males and 9% (6/66) of females reported seeking medical attention for a subsequent fall during the 60-day post discharge period. Of the seven who sought medical attention for a subsequent fall, all returned to an ED, and 71% (5/7) returned to the ED at which they had been treated for the initial fall injury.

#### Receipt of brochure

Thirty-two percent of participants (37% of males, 30% of females) recalled receiving the brochure providing contact information for a local falls prevention program (data not shown).

## Discussion

Older adult falls are increasing as a consequence of population aging and independent factors that are not fully understood (Cigolle et al. 2015). Thus, the number of older adults presenting at EDs with a fall-related complaint will invariably increase in the foreseeable future. Older people presenting to an ED for a fall injury are at high risk for subsequent falls (Sattin 1992; Campbell et al. 1989; Tinetti et al. 1988) and they do not generally receive the care they need to manage this risk. Accordingly, the ED visit for a fall presents an opportunity to mobilize health care resources around prevention of subsequent falls. Given the likelihood that an older adult presenting with a fall complaint at an ED is likely to fall again (Sattin 1992; Campbell et al. 1989; Tinetti et al. 1988), the physician possesses actionable knowledge that rarely translates into an adequate healthcare system response, often due to resource constraints in the ED setting. We found that while 71% of participants spoke with their provider regarding their fall, less than 35% engaged in falls prevention activities. Specifically, in regard to medications, vision, and community based falls prevention programs, less than half reported discussing these matters with their provider. This low percentage indicates that both the providers and patients need further education on the effectiveness of clinical and community fall prevention interventions. Our finding that only 42% of the subjects reported that they were “somewhat” or “very likely” to engage in a falls prevention program in the future highlights the importance of health care providers initiating conversations about fall prevention strategies. For many older adults, falls carry a stigma associated with declining capabilities and loss of independence. Additionally, many older adults are uninformed about effective falls prevention strategies or may lack confidence in their ability to prevent falls (Hill et al. 2011).

While our result indicate that older adults do not generally partake in falls prevention activities post-ED discharge, this study has several limitations. First, findings may not be generalizable to all patient populations because our study participants were drawn from a single, urban academic site that serves as a safety-net hospital. Second, we did not enroll non-English speaking patients which also limits generalizability, although there is extensive literature on falls incidence worldwide and many non-English speaking patients face the similar barriers to engaging in falls prevention. Third, participants may have experienced recall bias and thus may have had conversations about falls prevention but forgotten, though we tried to limit this by having a relatively short follow-up period. Alternatively, patients may have over-reported on their falls prevention/help seeking behaviors for two reasons. First, all patients received a Matter of Balance flyer as part of their discharge packet which could have latently encouraged such types of discussion. Second, patients may view an ED visit for a fall as a major event, and due to social desirability, may have reported discussing this fall with anyone on the follow-up questionnaire even though such discussions may have never taken place.

## Conclusions

Despite the availability of resources designed to help health care providers integrate fall prevention education into their practices, EDs are typically not equipped to conduct a falls risk assessment or intervention. Moreover, a large systematic review on the factors influencing the promotion of fall prevention programs suggests that the reasons for success and failure are complex and the promotion of an intervention must be tailored to the needs of the patient (Child et al. 2012). Therefore, further studies are required to assess ED-based interventions that motivate patients to engage in falls prevention activity, but are also practical given the busy, episodic, and often chaotic ED environment. Such interventions could include referral to a multidisciplinary falls risk assessment clinic, if such a service were available. In the meantime, ED providers attending older adult fall patients can, at a minimum, encourage their patients to talk to their primary care physicians and families about falls prevention and provide a list of local, evidence-based fall prevention programs to patients who might benefit from participation (Howland 2015), anticipating that this recommendation will be followed-up and assessed by their primary care physician.

## Additional file

Additional file 1: 60 Day Prospective Older Adult Falls Data Set and Data Dictionary. (XLSX 23 kb)

## Abbreviations

ED: Emergency department
EMR: Electronic medical record
MOB: Matter of balance
RA: Research assistant
ROI: Return on investment
